# Swimming in cold water increases the browning process by diminishing the Myostatin pathway

**DOI:** 10.1007/s11033-024-09586-3

**Published:** 2024-08-02

**Authors:** Niloofar Rahmani, Pezhman Motamedi, Sadegh Amani-Shalamzari, Kurt A. Escobar, Katsuhiko Suzuki

**Affiliations:** 1 Department of Exercise Physiology, Faculty of Physical Education and Sports Science, Kharazmi University, Tehran, Iran; 2 Physiology of Exercise & Sport Lab, Department of Kinesiology, California State University, Long Beach, USA; 3 Faculty of Sport Sciences, Waseda University, Mikajima, Tokorozawa Japan

**Keywords:** Exercise training, Cold stress, Browning, White fat

## Abstract

**Background:**

Brown adipose tissue (BAT) is a thermogenic tissue that uncouples oxidative phosphorylation from ATP synthesis and increases energy expenditure via non-shivering thermogenesis in mammals. Cold exposure and exercise have been shown to increase BAT and browning of white adipose tissue (WAT) in mice. This study aimed to determine whether there is an additive effect of exercise during cold exposure on markers related to browning of adipose tissue. in Wistar rats.

**Methods:**

Twenty-four male Wistar rats were randomly divided into three groups: Control (C, 25˚C), Swimming in Neutral (SN, 30˚C) water, and Swimming in Cold (SC, 15˚C) water. Swimming included intervals of 2–3 min, 1 min rest, until exhausted, three days a week for six weeks, with a training load of 3–6% body weight. After the experimental protocol, interscapular BAT and inguinal subcutaneous white adipose tissue (WAT) were excised, weighed, and processed for beiging marker gene expression.

**Results:**

SN and SC resulted in lower body weight gain, associated with reduced WAT and BAT volume and increased BAT number with greater effects observed in SC. Myostatin protein expression was lower in BAT, WAT, soleus muscle, and serum NC and SC compared to the C group. Expression of the interferon regulatory factor-4 (IRF4) gene in both BAT and WAT tissues was significantly greater in the SC than in the C. Expression of the PGC-1α in BAT was significantly increased in the SC compared to C and increased in WAT in NC and SC. Expression of the UCP1 in BAT and WAT increased in the SC group compared to other groups.

**Conclusion:**

The findings demonstrate that six weeks of swimming training in cold water promotes additive effects of the expression of genes and proteins involved in the browning process of adipose tissue in Wistar rats. Myostatin inhibition may possess a regulator effect on the PGC-1α – UCP1 pathway that mediates adipose tissue browning.

## Introduction

Adipose tissue is a complex organ that possesses several metabolic functions and includes several types. While white adipose tissue (WAT) primarily functions to store and release lipids dependent on energy demands, brown adipose tissue (BAT) is responsible for non-shivering thermogenesis through the uncoupling of oxidative phosphorylation from ATP synthesis [1, 2]. BAT are rich in mitochondria as well as uncoupling protein 1 (UCP1). UCP1 increases proton leak across the mitochondrial inner membrane releasing the proton motive force as heat rather than ATP synthesis through ATP synthase [2, 3]. The energy expenditure for thermogenesis in BAT has been considered a potential therapeutic target in the treatment of obesity due to it non-reliance on physical activity [2, 4]. Moreover, in addition to increasing BAT activity, WAT is capable of “browning” (i.e. beige adipose tissue) in response to various stressors. As a result BAT, may represent key role in the development and treatment of obesity. Accumulation of visceral and subcutaneous WAT is associated with metabolic disorders and diseases, decreasing WAT reduces the risk of such diseases [5]. Increasing BAT may contribute to the reduction in obesity and WAT through increasing energy expenditure and utilization of stored lipids for heat production. Therefore, interventions to enhance BAT and/or increase the “browning” of WAT (i.e. beige cells) have been explored including cold exposure and exercise.

BAT is a key tissue in thermogenesis and maintenance of body temperature in mammals. Inhabitants of cold and mountainous regions possess higher amounts of BAT and exposure to cold can increase BAT thermogenesis as well as cause conversion of some WAT to beige cells.

Cold-induced BAT activation is mediated by activation of the sympathetic nervous system (SNS) which release of norepinephrine (NE) activates of the β-adrenergic receptor (β-AR) [4]. In turn, cyclic adenosine monophosphate (cAMP) is activated [4] causing a signaling cascade in in BAT, resulting in the production of interferon regulatory factor 4 (IRF4) [6]. This cytokine mediates the UCP1 expression through interaction with peroxisome proliferator-activated receptor gamma coactivator 1-alpha (PGC1α) [6, 7]. IRF4 has been identified as a transcription partner for PGC-1 and a significant regulator of mitochondrial origin and thermogenesis in BAT. Mice lacking IRF4 in BAT are vulnerable to cold exposure and obesity and insulin resistance during high-fat diet (HFD), while mice that overexpress IRF4 in BAT have opposite phenotypes [8]. IRF4 has the potential to enhance mitochondrial function and enhance muscle exercise capacity which may involve the inhibition of myostatin. While myostatin is involved in the regulation of muscle mass, myostatin blockage has been demonstrated to increase the expression of UCP1 in BAT [7]. Acute inhibition of myostatin signaling using an ActRIIB inhibitor increases the weight of BAT and improves mitochondrial function, energy expenditure, and cold tolerance [9]. Additionally, increased serum myostatin has been observed in, mice exposed to high temperatures while exhibiting reduced exercise capacity [9]. Skeletal muscle appears to serve as the primary source of myostatin, however, BAT has been shown to play a significant role in serum myostatin levels and can secrete significant amounts of myostatin in response to blood warming [7].

Existing literature demonstrates that exercise induces morphological and biochemical changes in WAT to that of BAT [10, 11]. Exercise training promotes the browning WAT, either by stimulating the SNS and releasing NE during exercise [12] or by modification of myokines including myostatin [13]. Serum and muscle levels of myostatin are reduced by aerobic exercise [12]. Four weeks of swimming at normal and warm temperatures (35 °C) reduce myostatin levels in fast- and slow-twitch muscles [14]. It was also shown that suppression of myostatin with ActRIIB for 12 weeks reduced myostatin and increased browning in WAT in mice [13]. In addition, in previous studies, the effectiveness of swimming in water at normal and warm temperatures (36 –35 °C) has been demonstrated to increase BAT capacity and thermogenic activity in rats [15]. Swimming training for 6 to 8 weeks increased BAT mass and the thermogenic activity of BAT as well as UCP-1 expression in rodents [16–18], however some studies reported no overt effect of exercise on BAT activity [19, 20].

Considering that both cold stress and exercise are involved in BAT regulation and the browning of WAT, it has been speculated that their combination may have a synergistic effect through common or different mechanisms in WAT browning [21, 22]. For example, swimming in cold water is a stressful physiological condition that may magnify the body’s response to exercise [23] and beneficial adaptive changes in the body. In this regard, eight weeks of swimming in cool water (24 °C) have been reported to increase the expression of genes linked to mitochondrial biogenesis in adipose tissue of mice compared to the control conditions [24]. However, a study conducted by Da Silva et al. [25] reported that eight weeks of swimming in cool water (20 °C) did not enhance the browning of WAT and did not stimulate BAT thermogenesis [25]. It appears that colder water temperatures may be required to augment BAT. It should be noted that water temperatures in previous studies were not classified as cold temperatures [23]. Additionally, it appears that exercise combined with cold exposure may be an effective method to regulate the myokine profile. Water temperature and exercise training can provide different acclimatization, but their interactive effects are poorly documented. Hence, we postulated that swimming in cold water (15 °C) would elicit greater BAT and WAT browning gene expression and serum myostatin alterations compared swimming in tepid water in Wistar rats. Therefore, the objective of this research is to determine if there is a synergistic effect on the genes involved in the browning process following six weeks of cold swimming through the PGC1α/IRF4/myostatin pathway.

## Materials

### Animals

Twenty-four male Wistar rats (3 months old) were purchased from the Pasteur Institute and housed in conventional cages with a 12-h:12-h light/dark cycle in typical temperatures (∽ 25˚ C). In two weeks of adaptation phases, animals swam in tepid and cold water for 20 min (one-minute intervals, one week cold, one week tepid water). Then, animals were randomly divided into three groups: Control (C, *n* = 8), Swimming in Neutral water (SN, *n* = 8), and Swimming in Cold water (SC, *n* = 8). The laboratory temperature was 25 ± 2 °C. The tepid water was 30 ± 2 °C, while water temperature in Cold water condition was 15 ± 1 °C. To display the desired temperature, a waterproof digital thermometer was placed in the water. During the six-week training intervention, rats were allowed ad libitum access to water and standard commercial chow. Rat’s body weights were measured weekly by digital scales. It is worth mentioning that this study has adopted the same methods as our previous study [10] but with different purposes and parameters.

### Swimming to exhaustion test

Following the last training session, a progressive swimming test was conducted to assess the aerobic level of the animals, as described in the Almeida et al. study [26]. The trial included three-minute swimming intervals separated by a one-minute break. In the first three stages, 1, 2, and 3% of their body weight as an external burden were attached to the rat’s tail, respectively. Then, in the next steps, each attempt, the external load increases by 0.5% of body weight until exhausted. The number of swimming repetitions was used for statistical analysis.

### Exercise protocol

The animals swam into the glass aquarium with a length of 100 cm width, and a water depth of 50 cm. The swimming protocol consisted of intervals of two minutes until exhaustion, separated by one minute of rest; three times per week for six weeks. An initial external load was 3% of the rat’s body weight attached to its tail; then it increased by 1% if the rats could swim ten repetitions successfully. Moreover, if they swam ten successful repeats with 6% of their body weight, the swimming time was increased to three minutes.

### Blood and tissue sampling

Following the intervention, the animals were anesthetized using cervical dislocation; blood samples were collected intracardially and centrifuged for 10 min at 4000 rpm; then the serum was stored at -20 °C. The interscapular BAT, subcutaneous WAT slices, and soleus muscle were excised and divided into two sections. A sample of both adipose tissues was fixed in 10% formaldehyde, passaged, and embedded in paraffin, and a slice was frozen in nitrogen and stored at -80 °C for further analysis.

### Histological analysis

The adipose tissues were put in 10% paraformaldehyde, then dehydrated by alcohol gradient concentrations, cleaned with xylene solvent and incorporated into the paraffin. The paraffin blocks were sectioned by a microtome (Leica, Germany) at 5–10 μm, mounted on slides, deparaffinized, and stained with hematoxylin and eosin based on instructions. The stained tissue samples were visualized using a light microscope (Nikon, Tokyo, Japan); then, the photos were examined using Image J software. A graduated lens and appropriate measurement software, such as Dyno Capture or Image G (Fiji), were used to measure the average diameter of fat cells. The histomorphometric parameter was calculated using 6 figures (*n* = 6) for each group. The number of adipocytes was counted in a 1 mm^2^ area in every tissue section.

### Protein assay

Using a special buffer containing 50 mM Tris-HCI (pH 7.8), 2 mM potassium phosphate, 2 mM EDTA, 2 mM GTA, 10% glycerol, 1% Triton X-100, 1 mM dithiothreitol, 3 mM benzamidine, 1 mM sodium orthovanadate, 10 mM leupeptin, 5 mg/ml aprotinin, and 1 mM 4 benzenesulfonyl fluoride, 100 mg of frozen samples were homogenized. The homogenates were then centrifuged at 12 000 g for 20 min at 4 °C, the supernatant was eliminated, and protein concentrations were determined using the Bio-Rad protein assay. Myostatin concentrations (RK03823, Sensitivity was 31.1 pg/mL, Zell Bio, Germany) were measured by specific kits. Serum norepinephrine concentrations were measured by a specific kit (ZB-11,137 C, Zell Bio, Germany).

### Gene expression

Total RNA was extracted from 100 mg of adipose tissue with Trizol solution according to the instructions (Invitrogen). Initially, the purity and quantity of RNA were determined by spectrophotometry with a NanoDrop ND-1000 (VWR, Radnor, PA, USA). A Qiagen cDNA synthesis kit (cat: K1622) was used for cDNA synthesis. The relative expression of mRNA was determined by QRT-PCR using SYBR Green dye. The thermal cycle schedule was 94 °C for 3 min, followed by 30 94 °C cycles for 0.5 min, 54 °C for 1 min, and 72 °C for 0.5 min. GAPDH mRNAs were used as a standardized gene. Table 1 shows the forward and reverse sequences of primers. The 2^−∆∆CT^ formula determined the fold change expression.

**Table 1:** The forward and reverse sequences of gene primers

Gene name	Forward sequence	Reverse sequence
IRF4	TGGCAGTTGGGGTATCATGTCTT	ACAGCAACAACAACAACAACAGCA
PGC1α	AACAAGCACTTCGGTCATCC	CTTCGCTGTCATCAAACAGG
UCP1	GCCTCTACGATACGGTCCAA	CTGACCTTCACCACCTCTGT
GAPDH	AGGTCGGTGTGAACGGATTTG	TGTAGACCATGTAGTTGAGGTCA

### Statistical analysis

The data was analyzed using SPSS, IBM, version 19. The descriptive data were reported by mean ± standard deviation (SD). To define the main effects of interventions on the variables, a one-way analysis of variance (ANOVA) was performed. If significant results were found, the Tukey post-hoc test was used. To discern the magnitude and direction of the linear relationship between variables, the bivariate Pearson correlation coefficient (r) was calculated. The significance level was considered at *p* ≤ 0.05 to accept the main effects.

## Results

Body weight of animals was different pre- and post-intervention between groups. C, SN, and SC groups body weight increases, were 29.6% (257 ± 9 to 333 ± 5 g), 18.1% (258 ± 9 to 305 ± 16 g), and 7.9% (254 ± 12 to 274 ± 9 g), respectively. Table 2 presents the diameter and number of BAT and WAT cell number. There was no significant difference in WAT cell count between the groups (F = 1.56, R^2^ = 0.13, *p* = 0.233). However, the diameter of WAT cells differed between groups (F = 69.64, R^2^ = 0.87 *p* = 0.001). The post-hoc Tukey test showed the volume of WAT cells in the C group was significantly higher than in the other groups (*p* < 0.001). Furthermore, the volume of WAT cells in the SC group differed significantly from the SN groups (*p* = 0.007).

**Table 2:** Diameter of adipose tissues, incremental test, and norepinephrine level in groups

	C	SN	SC
Brown diameter (µm/mm^2^)	38.25 (1.79)	26.31 (2.82)^a^	16.85 (2.01)^a, b^
Brown adipose count (n/mm^2^)	18.87 (2.90)	31.00 (1.77)^a^	33.75 (2.05)^a^
White diameter (µm/mm^2^)	50.76 (4.56)	37.45 (1.77)^a^	31.83 (2.93)^a, b^
White adipose count (n/mm^2^)	11.12 (0.95)	11.25 (2.19)	12.75 (1.98)
Incremental test (n)	4.63 (0.74)	9.00 (0.54)^a^	7.25 (0.88)^a, b^
Norepinephrine (ng/ml)	2.32 (0.20)	2.64 (0.19)	2.95 (0.62)^a^

C: control group; EC: exposure to a cold group; SN: Swimming in Neutral water group; SC: Swimming in cold water group; a significant difference with the C group; b significant differences with the SN group

The diameter (F = 181.7, R^2^ = 0.95, *p* < 0.001) and number (F = 95.34, R^2^ = 0.90, *p* < 0.001) of BAT cells have also differed significantly between groups. Post-hoc Tukey test showed there were significant differences in BAT cell number and diameter between the C group and both trained groups (*p* < 0.001). Moreover, there was a significant difference in BAT volume between the SC and SN groups. We observed a significant positive correlation between the percentage change in rat’s body weight and the volume of WAT (*r* = 0.75, *p* = 0.001) and BAT (*r* = 0.78, *p* = 0.001) cells. Also, there was a significant negative correlation between the percentage change in body weight and the number of BAT cells (*r*=-0.79, *p* = 0.001), but not significant with the number of WAT cells (*r*=-0.35, *p* = 0.097).

The results of the incremental swimming test showed that there was a significant difference between groups in swimming intervals to exhausting (F = 71.62, R^2^ = 0.87, *p* = 0.001). The post-hoc Tukey test showed both trained groups had a higher swimming capacity than the control group (*p* < 0.001). In addition, the SN group demonstrated a higher swimming capacity compared to the SC group (*p* = 0.001).

We observed a significant difference in serum NE levels between groups as shown in Table 2 (F = 5.08, R^2^ = 0.33, *p* = 0.016) The NE values for the SC group were significantly higher compared to the C group (*p* = 0.012).

**Fig. 1:**
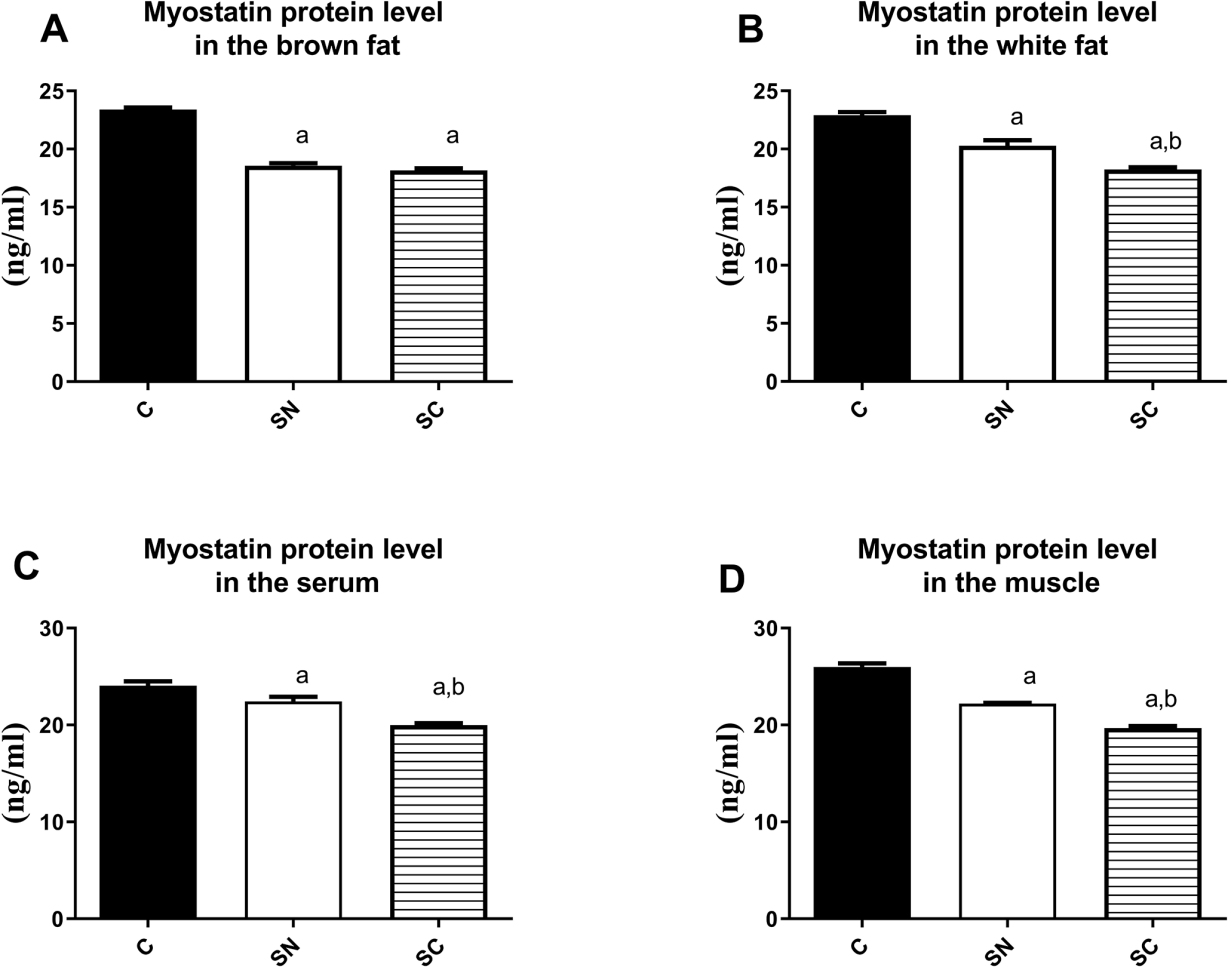
Level of myostatin in the selected tissues in the groups. C: control, SN: Swimming in Neutral, and SC: Swimming in Cold water. **a** significant difference with the C group; **b** significant differences with the SN group

The result of ANOVA showed there were significant differences in the myostatin levels in WAT (F = 49.57, R^2^ = 0.83 *p* < 0.001), BAT (F = 252.5 R^2^ = 0.96 *p* < 0.001), serum (F = 27.21 R^2^ = 0.72 *p* < 0.001), and muscle (F = 159.8 R^2^ = 0.94 *p* < 0.001) as presented in Fig. 1. In BAT, the post-hoc Tukey test showed that the amount of myostatin protein in both SN and SC groups was significantly reduced relative to the C group (*p* < 0.01). In the WAT, serum, and muscle, the significant differences were between the SC group and SN and C groups (*p* < 0.001) and also between the SN group and the C group (*p* < 0.05).

**Fig. 2:**
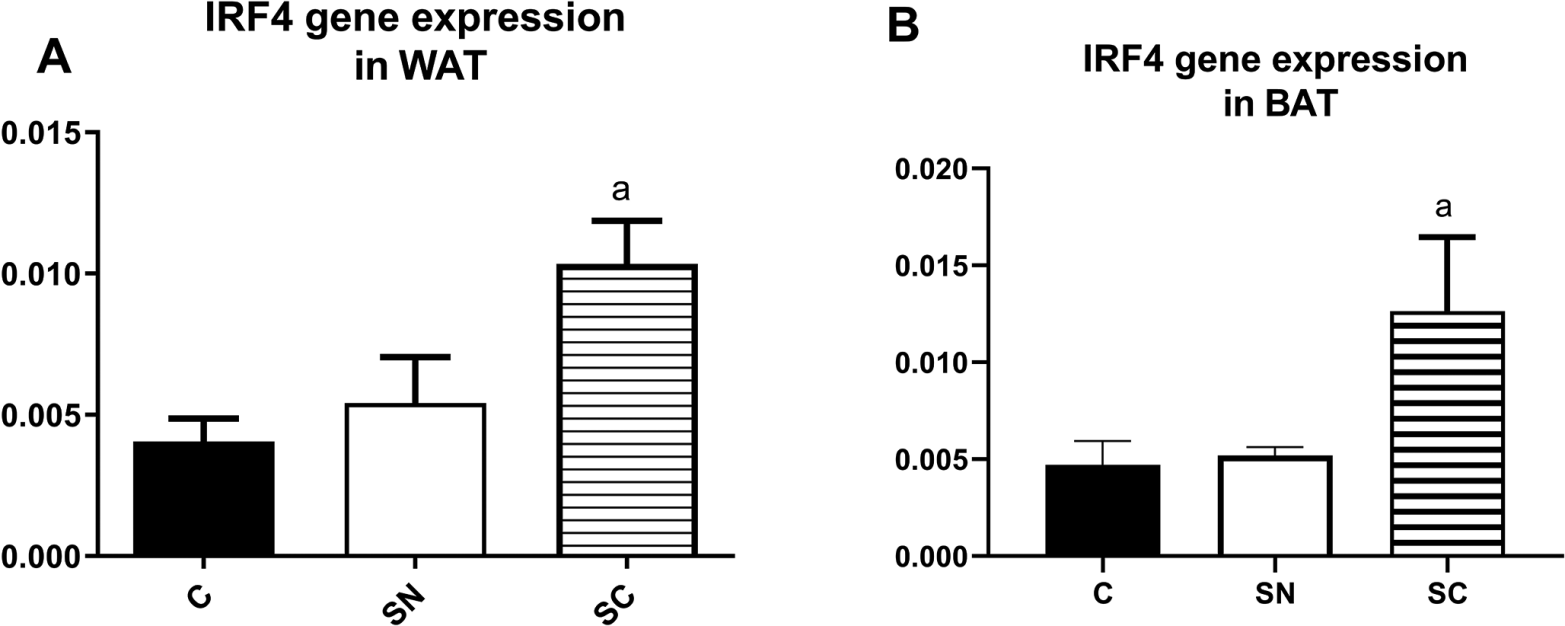
The mean (SD) expression of the IRF-4 mRNA in the brown and white fat tissues. C: control, SN: Swimming in Neutral, and SC: Swimming in Cold water. a significant difference with the C group

Figure 2 shows the IRF4 mRNA expression was significantly different between groups in WAT (F = 5.85, R^2^ = 0.66, *p* = 0.017) and BAT (F = 4.01, R^2^ = 0.40, *p* = 0.046). The post-hoc test showed that an increase in IRF4 gene expression in the SC group significantly differed from the C group in WAT and BAT (*p* < 0.05).

**Fig. 3:**
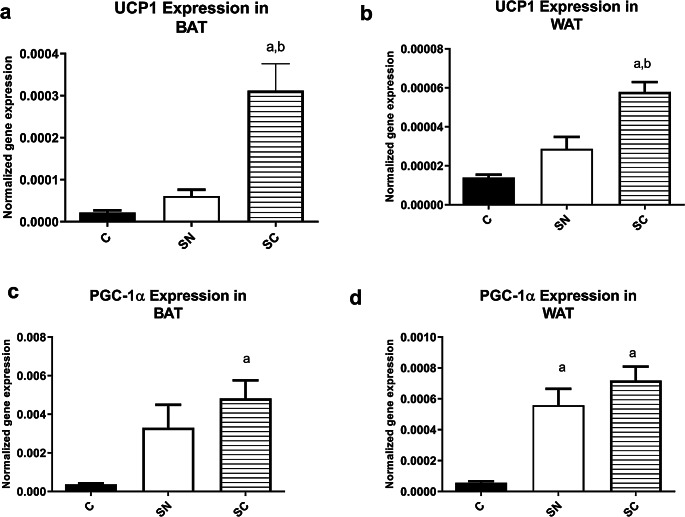
The mean (SD) expression of the UCP1 (**a** and **b**) and PGC-1α mRNA (**c** and **d**) in the brown and white fat tissues. C: control, SN: Swimming in Neutral, and SC: Swimming in Cold water. a significant difference with the C group; b significant differences with the SN group

Figure 3 displays that the UCP1 mRNA expression was significantly different between groups in WAT (F = 23.09, R^2^ = 0.69, *p* = 0.001) and BAT (F = 17.24, R^2^ = 0.62 *p* = 0.001). The post-hoc test showed that there were significant differences in both BAT and WAT between SC and SN and C groups (*p* < 0.05).

There were significant differences in PGC-1α mRNA levels in WAT (F = 18.44, R^2^ = 0.64 *p* < 0.001) and BAT (F = 6.73, R^2^ = 0.39 *p* = 0.005), as shown in Fig. 3c & d. In the BAT, the post-hoc test demonstrated that the expression of PGC-1α mRNA levels in the SC group was significantly different than C group (*p* = 0.004). In the WAT, the upregulation of the PGC-1α in both experimental groups was significantly different from the C group (*p* < 0.001).

## Discussion

The primary purpose of this study was to examine the effect of cold exposure and exercise independently and combined using cold water swimming on genes and protein expression related BAT and browning of WAT in Wistar rats including the PGC1α-IRF4-myostatin pathway. In addition to increasing exercise capacity (swimming to exhaustion)swimming in neutral temperature (SN) water increased PGC-1α expression in WAT and significantly decreased myostatin in WAT, BAT, muscle, and serum. Additionally, this intervention led to morphological changes in adipocytes, by reducing the BAT and WAT diameters and increasing the number of brown fat cells. However, SN failed to trigger the process of thermogenesis in BAT. Cold water swimming (SC) group had significantly lower WAT and BAT diameters and an increase in BAT numbers. These changes were associated with greater expression IRF4, UCP1, and PGC-1α in adipocytes and decreased myostatin in WAT, BAT, serum, and muscle. These results may indicate that exercise during cold exposure provides a synergistic effect on the activation of the browning and thermogenic process of adipose tissues by increasing IRF4 signaling cascade and reducing myostatin.

The observed effects of cold exposure and exercise appear to be mediated by norepinephrine which results in the activation of the β-adrenergic receptor (β-AR). The β3-adrenergic receptor is critical for cold-induced transcriptional activation of BAT and beige fat [27]. These signaling pathways stimulate cyclic adenosine monophosphate (cAMP) [4] and induce a signaling cascade in BATcells, resulting in the production of IRF4, a critical regulator of the biogenesis of mitochondria and thermogenesis in BAT. Interacting with PGC1α, IRF4 induces the expression of UCP1, a mitochondrial protein that uncouples proton motive force crossing the mitochondrial inner membrane through ATP synthase for ATP synthesis to heat production [6]. In this study, the expression of the genes PGC-1α was significantly increased in WAT in both swimming groups, however, only SC increased PGC-1α in BAT IRF4 expression was only significantly different in the SC group in BAT and WAT relative to the C group.

Expression of UCP1 gene in BAT and WAT in the SC group was significantly higher compared to SN and C groups… These data indicate while swimming exercise in tepid water conditions can elicit increases in genes related to browning of adipose tissue including WAT, exposure to cold during acute exercise results in greater gene activity including increases in UCP1 gene expression in BAT. Importantly, UCP1 is the protein responsible for the uncoupling of energy production and substrate use in oxidative phosphorylation for thermogenesis in BAT and “beige” adipose tissue, WAT that has developed BAT-thermogenic properties. In agreement with our findings, Aldis et al. [28]. also reported increased PGC-1α gene expression in BAT and WAT with no significant change in UCP1 mRNA [28]. It appears that WAT’s UCP1 response to exercise requires cold stress [10]. while in BAT, exercise training can activate the genes involved in thermogenesis (UCP1) [28]. In general, due to the greater of UCP1 and IRF4 in the SC group, it appears that the expression of UCP1 is highly correlated with IRF4 and IRF4 is another key indicator of thermogenesis.

Various myokines released by skeletal muscle during exercise are implicated in the browning process adipose tissue including myostatin [13]. Previous work has demonstrated blocking myostatin increases the expression of UCP1 in BAT and cold exposure and regular exercise reduce myostatin levels in skeletal muscle and serum [6, 9, 29]. Moreover, Kong et al. [7] demonstrated that increased expression of IRF4 in BAT was associated with a decrease in myostatin protein in serum and its gene expression in BAT but not in muscle [30]. In addition, by blocking IRF4 in BAT increased myostatin protein level in both BAT and serum while remaining unchanged in the muscle. However, there are still uncertainties about the interplay of myostatin between fat and muscle tissue. To investigate the relationship between between these tissues, myostatin levels we measured myostatin protein expression in soleus muscle, BAT, WAT, as well as serum. We observed a significantly lower expression of myostatin protein in BAT in the SC group compared to the SN and C groups. Expression of myostatin in WAT was also lower in SN and SC groups compared to the C group. However, SC group myostatin levels were significantly lower compared SN group in WAT. In addition, serum myostatin concentrations were significantly lower in the exercise groups (SC and SN) compared to the C group. These alterations in myostatin amongst measured tissues in response to cold exposure and exercise appear to relate to changes IRF4. Altogether, our data suggest cold exposure enhances inhibition of myostatin expression compared to exercise alone and may relate to gene activity involved in BAT and browning of WAT.

Related to exercise performance, both swimming groups (SC and SN) demonstrated higher swimming capacity to exhaustion compared to C animals. Independent of water temperature, rodent studies have reported that swimming training improves aerobic ability and prolonged exercise time [18, 20]. However, swimming in cold water elicits a greater metabolic and thermoregulatory stress compared to neutral water temperature [31]. , Consequently, the swimming time to exhaustion is reduced due to greater energy expenditure contributing to non-shivering thermogenesis in BAT rather than muscular activity to support exercise [9, 29, 32]. Therefore, while BAT and beige adipose tissue activity during exercise may be enhanced with cold exposure, a consequent reduction in exercise capacity can be observed.

We admit that there were some limitations in our investigation. The first was the lack of access to laboratory equipment to fullu measure rat body composition. Second, the lack of funding to count the number of mitochondria that is important to interpret the outcomes. Therefore, we suggest that researchers conduct a similar study to measure the interaction effect of cold water swimming on mitochondrial counts and proteins involved in mitochondrial biogenesis. And lastly, further investigation of the implications of BAT on energy expenditure and obesity in humans should be conducted as currently there are conflicting data on this topic in humans [3, 33, 34].

### Conclusion and implication

Collectively, our results showed that swimming in tepid and cold water had a positive impact on body weight management through changes in adipose tissue. However, the most significant decrease in the diameter of the fat cells (WAT and BAT) and the increase in the number of brown fat cells were observed during the SC intervention. These changes were associated with overexpression of the IRF4, UCP1, and PGC-1α genes in adipocytes and decreased myostatin in WAT, BAT, and muscle, which shows swimming in cold water has a synergistic effect on the activation of the browning and thermogenic process. Overall, due to the results in the SC group with lower swimming volume, interval swimming in cold water is recommended for weight loss.

## Data Availability

If requested, it will be available.
